# Endoscopic ultrasound-guided gastroenterostomy to treat obstructive gastric twist after laparoscopic sleeve gastrectomy

**DOI:** 10.1055/a-2333-9183

**Published:** 2024-07-08

**Authors:** Laurent Monino, Yannick Deswysen, Maximilien Thoma, Pierre H. Deprez, Tom Moreels

**Affiliations:** 1 Department of Gastroenterology & Hepatology, Cliniques universitaires Saint-Luc, Université catholique de Louvain, Brussels, Belgium; 2 Department of Digestive Surgery, Cliniques universitaires Saint-Luc, Université catholique de Louvain, Brussels, Belgium

Sleeve gastrectomy is the number one bariatric surgical intervention worldwide to treat morbid obesity. The rate of gastric stenosis after sleeve gastrectomy is around 2 to 4% 1 2 . A gastric twist represents a functional gastric stenosis. Endoscopic management with pneumatic dilation or stent is proposed as first-line therapy 1 2 3 . In case of failure, a surgical conversion to Roux-en-Y gastric bypass (RYGB) is performed. Endoscopic ultrasound-guided gastroenterostomy (EUS-GE) using an oroenteric catheter is a new approach to treat a benign gastric outlet obstruction (GOO) 4 5 . We report the case of a patient with a gastric twist after laparoscopic sleeve gastrectomy successfully treated with EUS-GE after failure of repeat endoscopic dilatation.

A 69-year old woman underwent sleeve gastrectomy. One month later, she presented symptoms of GOO with a gastric outlet obstruction scoring system (GOOSS) score of 1. Endoscopy showed peptic esophagitis associated with a mid-gastric twist ( Fig. 1 **a, b** ) confirmed by computed tomography scan ( Fig. 1 **c** ). Three sessions of endoscopic dilatation were performed without clinical improvement. An EUS-GE was proposed to “bypass” the mid-gastric twist ( Video 1 ). An oroenteric catheter was placed over a guidewire to fill the jejunal lumen. Next, the target jejunal limb was identified by EUS and punctured with the electrocautery-enhanced lumen-apposing metal stent (LAMS) in pure cut mode. The LAMS was deployed connecting the gastric and jejunal lumen without adverse events. Clinical improvement with a GOOSS score of 3 was reported and confirmed by radiology and endoscopy at 1 and 3 months ( Fig. 2 **a, b** ).

**Fig. 1:**
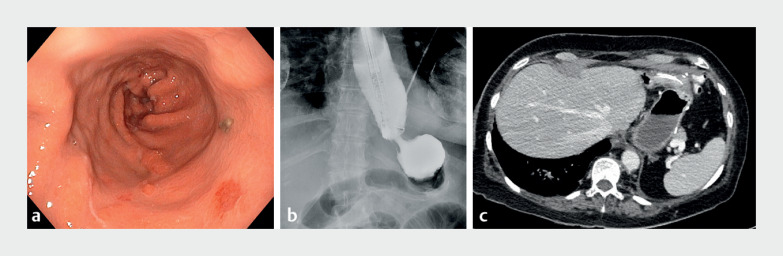
Diagnosis of the gastric twist after sleeve gastrectomy. **a** Twist of the stapling line. **b** Esophageal dilation and distal gastric obstruction confirmed with endoscopic contrast injection. **c** Gastric outlet obstruction due to the gastric twist.

Endoscopic ultrasound-guided gastroenterostomy using wireless endoscopic simplified technique with oroenteric drain to treat gastric outlet obstruction due to gastric twist after sleeve gastrectomy.Video 1

**Fig. 2:**
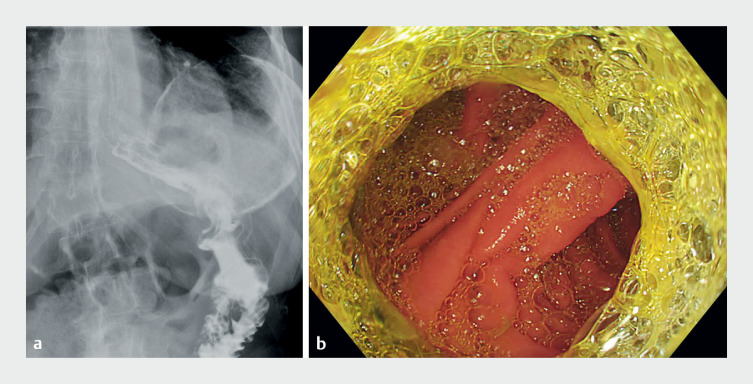
Endoscopic and radiological imaging 3 months after endoscopic ultrasound-guided gastroenterostomy. **a** Contrast injection bypassing the gastric twist. **b** End-to-side gastroenterostomy with lumen-apposing metal stent.

The management of a gastric twist with clinical implications after sleeve gastrectomy is challenging. The improved technical and clinical success of EUS-GE has allowed it to be used in case of a benign GOO due to gastric twist. Moreover, EUS-GE avoided surgical conversion to RYGB. Future studies are needed to define what to do with the LAMS in case of benign gastric outlet obstruction: remove it, replace it, or leave it.

Endoscopy_UCTN_Code_TTT_1AS_2AK

## Notes

Endoscopy E-Videos https://eref.thieme.de/e-videos
*E-Videos* is an open access online section of the journal *Endoscopy* , reporting on interesting cases and new techniques in gastroenterological endoscopy. All papers include a high-quality video and are published with a Creative Commons CC-BY license. Endoscopy E-Videos qualify for HINARI discounts and waivers and eligibility is automatically checked during the submission process. We grant 100% waivers to articles whose corresponding authors are based in Group A countries and 50% waivers to those who are based in Group B countries as classified by Research4Life (see: https://www.research4life.org/access/eligibility/ ). This section has its own submission website at https://mc.manuscriptcentral.com/e-videos .
